# Peroxisome Proliferator-Activated Receptor-Gamma Agonists Suppress Tissue Factor Overexpression in Rat Balloon Injury Model with Paclitaxel Infusion

**DOI:** 10.1371/journal.pone.0028327

**Published:** 2011-11-29

**Authors:** Jun-Bean Park, Baek-Kyung Kim, Yoo-Wook Kwon, Dominik N. Muller, Hyun-Chae Lee, Seock-Won Youn, Young-Eun Choi, Sae-Won Lee, Han-Mo Yang, Hyun-Jai Cho, Kyung Woo Park, Hyo-Soo Kim

**Affiliations:** 1 Department of Internal Medicine, Seoul National University College of Medicine, Seoul, Korea; 2 National Research Laboratory for Stem Cell Niche, Seoul National University College of Medicine, Seoul, Korea; 3 Innovative Research Institute for Cell Therapy, Seoul National University Hospital, Seoul, Korea; 4 Max-Delbruck Center and Experimental and Clinical Research Center, Berlin, Germany; 5 Molecular Medicine and Biopharmaceutical Sciences, Seoul National University, Seoul, Korea; University of Modena and Reggio Emilia, Italy

## Abstract

The role and underlying mechanisms of rosiglitazone, a peroxisome proliferator-activated receptor-gamma (PPAR-γ) agonist, on myocardial infarction are poorly understood. We investigated the effects of this PPAR-γ agonist on the expression of tissue factor (TF), a primary molecule for thrombosis, and elucidated its underlying mechanisms. The PPAR-γ agonist inhibited TF expression in response to TNF-α in human umbilical vein endothelial cells, human monocytic leukemia cell line, and human umbilical arterial smooth muscle cells. The overexpression of TF was mediated by increased phosphorylation of mitogen-activated protein kinase (MAPK), which was blocked by the PPAR-γ agonist. The effective MAPK differed depending on each cell type. Luciferase and ChIP assays showed that transcription factor, activator protein-1 (AP-1), was a pivotal target of the PPAR-γ agonist to lower TF transcription. Intriguingly, two main drugs for drug-eluting stent, paclitaxel or rapamycin, significantly exaggerated thrombin-induced TF expression, which was also effectively blocked by the PPAR-γ agonist in all cell types. This PPAR-γ agonist did not impair TF pathway inhibitor (TFPI) in three cell types. In rat balloon injury model (*Sprague-Dawley* rats, n = 10/group) with continuous paclitaxel infusion, the PPAR-γ agonist attenuated TF expression by 70±5% (n = 4; *P*<0.0001) in injured vasculature. Taken together, rosiglitazone reduced TF expression in three critical cell types involved in vascular thrombus formation via MAPK and AP-1 inhibitions. Also, this PPAR-γ agonist reversed the paclitaxel-induced aggravation of TF expression, which suggests a possibility that the benefits might outweigh its risks in a group of patients with paclitaxel-eluting stent implanted.

## Introduction

Peroxisome proliferator-activated receptor-gamma (PPAR-γ) agonists were developed originally for the treatment of diabetes but these agents also have been known to have potential cardio-protective effects [1], [2], [3], [4]. However, Nissen, in his meta-analysis, reported that rosiglitazone, a PPAR-γ agonist, was associated with increased risk of myocardial infarction [5]. On the other hand, several studies suggested that this PPAR-γ agonist did not adversely affect overall cardiovascular outcome [6], [7]. After years of debate, the US Food and Drug Administration announced the outcomes from its Advisory Committee hearing on rosiglitazone; a tightening of restriction on its use. Furthermore, this PPAR-γ agonist has been suspended in Europe and New Zealand, following a recommendation from the European Medicines Agency, and the suspension will remain in place unless conclusive data are provided that identify a certain group of patients in whom the benefits of this agent outweigh its risks.

However, there has still been ambiguity about the rosiglitazone-associated myocardial infarction. Anti-diabetic drugs are difficult to evaluate for the risk of cardiovascular events, since diabetic patients tend to experience cardiac events anyway. Thus, we are not sure if cardiovascular events are a consequence of the drug or a coincidence. Furthermore, post-hoc analysis of the Bypass Angioplasty Revascularization Investigation in Type 2 Diabetes (BARI 2D), presented at the American Diabetes Association 2010 Scientific Sessions, showed that this PPAR-γ agonist could reduce the risk of myocardial infarction in patients with coexisting diabetes and established coronary artery disease [7]. Intriguingly, the key difference of this study, compared with previous ones, might be the patient population which included more patients with comorbid heart disease. It is implausible that more thrombosis-prone condition with suspected culprit is related to having less thrombotic complication including myocardial infarction. It also suggests that the benefits of this agent might outweigh its risks in a certain group of patients. More importantly, contrary to expectations, there are no obvious results that can explain the possible pathogenesis of rosiglitazone-induced myocardial infarction. This is even in contrast to congestive heart failure, another worrisome complication of PPAR-γ agonists, to which an increase in plasma volume is suggested as the main culprit [8].

It is generally accepted that tissue factor (TF) contributes to the initiation and propagation of thrombus formation [9], [10], [11]. TF expression is up-regulated in several cell types within atherosclerotic plaques, namely, endothelial cells (ECs), monocytes and vascular smooth muscle cells (VSMCs). When the plaque is ruptured, TF is exposed to the bloodstream and coagulation cascade is triggered. In this context, TF plays a critical role in the development of acute coronary syndrome [12]. Paclitaxel and rapamycin, the major drugs that are widely used for drug-eluting stents, can induce TF overexpression [13], [14] and thus, TF has an important contribution to pathogenesis of stent thrombosis of drug-eluting stents [12].

For those reasons, the aim of the present study was to test the hypothesis that rosiglitazone has effect on TF expression in ECs, monocytes and VSMCs which are main cell types participating in vascular thrombus formation. We demonstrated that rosiglitazone suppressed TF overexpression, providing a possible explanation for the association between this PPAR-γ agonist and thrombotic complication. We also elucidated the underlying mechanism through detailed analyses.

## Results

### The PPAR-γ agonist Inhibits TF Expression in Human Umbilical Vein Endothelial Cells (HUVECs), Human Acute Monocytic Leukemia Cell Line (THP-1), and Human Umbilical Arterial Smooth Muscle Cells (SMCs)

**Figure 1:**
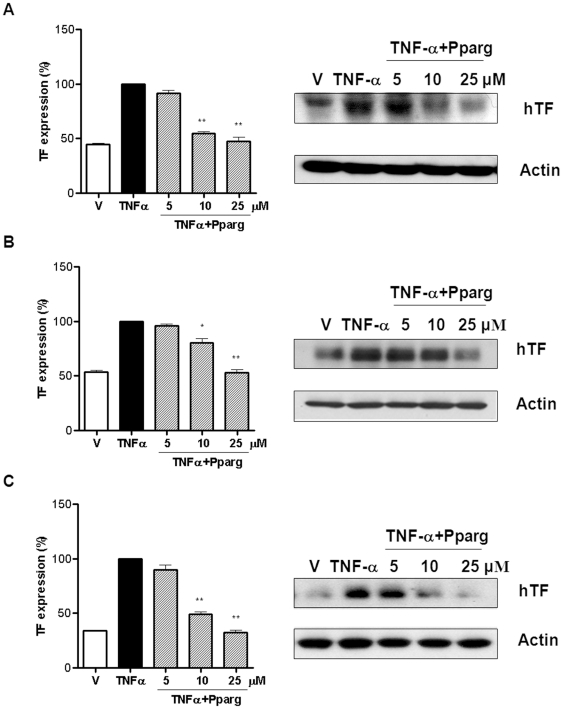
PPAR-γ agonist inhibited TF expression in ECs, monocytes and SMCs. (**A**) The PPAR-γ agonist suppressed TNF-α-induced TF expression in HUVECs in a concentration-dependent manner. (**B–C**) The PPAR-γ agonist also decreased TF expression in THP-1 (B) and SMCs (C). Average values of 3 different experiments. Data are presented as mean ± SEM. Values are given as percent of stimulation with TNF-α alone. *P = 0.002, **P<0.0001 vs TNF-α alone. Each number in western blot analysis indicates the concentration of the PPAR-γ agonist; all blots were normalized to actin expression. V = vehicle; Pparg = PPAR-γ agonist; hTF = human TF. See Figure S1 and S2 for the quantitative data and the effects of MAPK inhibitors on TF expression, respectively.

Since ECs, monocytes and VSMCs are the most essential cells that participated in intravascular thrombosis or stent thrombogenicity [13], we evaluated the effect of the PPAR-γ agonist on TF expression in these cell types. TF expressions were increased dramatically by TNF-α in all kinds of cells. After cells were pretreated with the PPAR-γ agonist (0, 5, 10, and 25 µmol/L), TF induction by TNF-α was inhibited in a concentration-dependent manner in all cell types (n = 3; *P*<0.0001 for TNF-α alone versus 25 µmol/L PPAR-γ agonist in all cell types; Figure 1A-C).

### The PPAR-γ agonist Suppresses Mitogen-activated protein Kinase (MAPK) Activation

**Figure 2:**
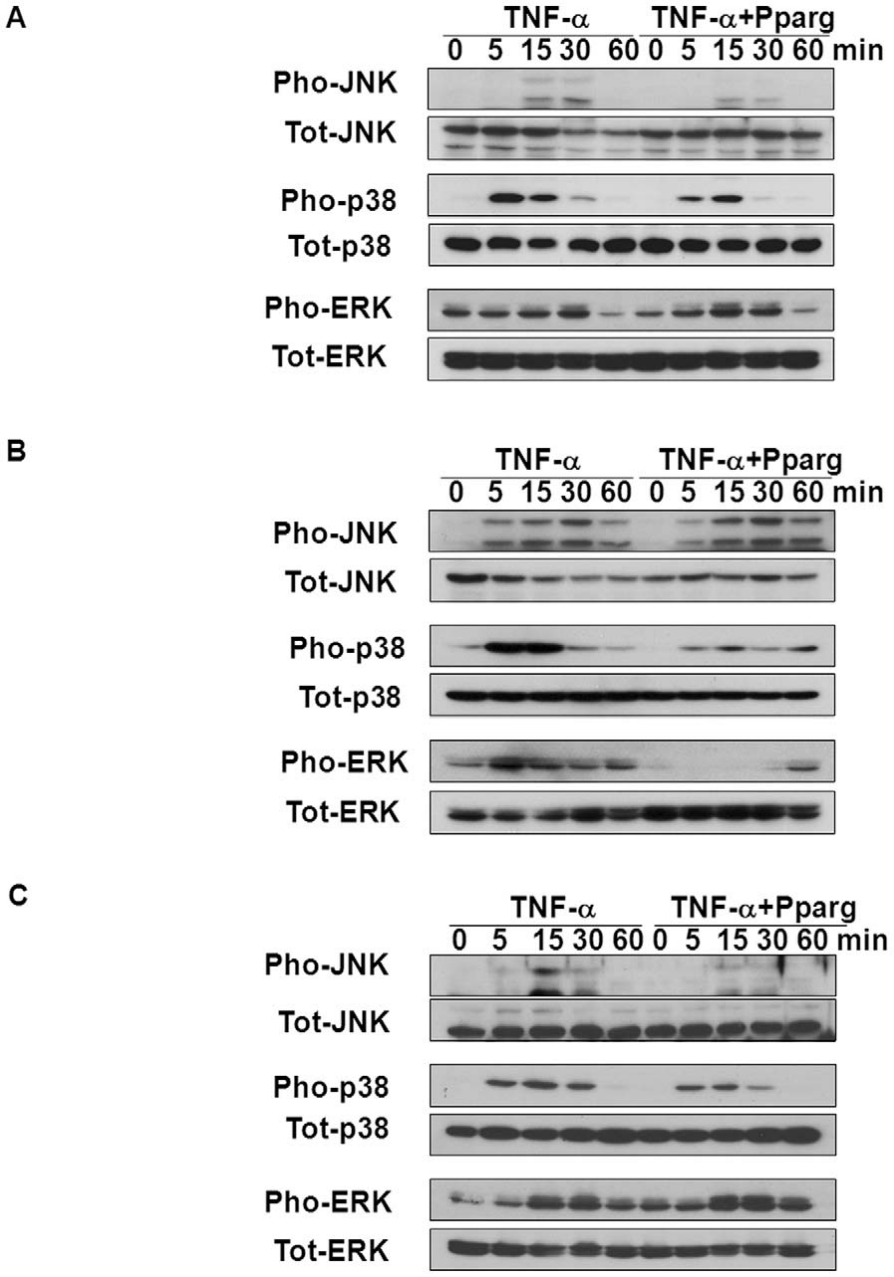
Cell type-specific inhibition of MAPK phosphorylation. (**A–C**) The overexpression of TF was mediated by the increased phosphorylation (Pho) of MAPK, which was blocked by the PPAR-γ agonist. The main MAPK varied depending on each cell type; HUVECs were dependent on JNK and p38 (A); THP-1 cells were dependent on p38 and ERK (B); SMCs were dependent on JNK and p38 (C). Total (Tot) levels of MAPK remained unchanged.

Because MAPK pathways were involved in generation or activation of various mediators that induced TF expression [12], we investigated the effects of the PPAR-γ agonist on MAPKs and its different effects on each cell type. First, we examined HUVECs at different time points (0, 5, 15, 30, 60 min) after stimulation with TNF-α. Phosphorylated forms of JNK, p38 and ERK were transiently increased by TNF-α. Treatment with the dose of 25 µmol/L of PPAR-γ agonist inhibited phosphorylation of JNK by 31±2%, p38 by 10±2% while leaving that of ERK unchanged (n = 3; *P*<0.0001, *P* = 0.009 respectively; Figure 2A, Statistical graphs are shown in Figure S1A). Like HUVECs, transient activations of MAPKs were observed after stimulation in THP-1 and SMCs. The PPAR-γ agonist suppressed phosphorylation of p38 by 48±2% and ERK by 43±6% in THP-1 (n = 3; *P*<0.0001, *P* = 0.001 respectively; Figure 2B, Statistical graphs are shown in Figure S1B) and JNK by 39±3% and p38 by 27±7% in SMCs (n = 3; *P*<0.0001, *P*<0.05 respectively; Figure 2C, Figure S1C). To confirm the specific effects of MAPKs on the process of TF expression under our experimental conditions, we performed western blotting after treating MAPKs inhibitors. TF expression was reduced by SP600125, SB203580, and PD98059, which specifically inhibit JNK, p38, and ERK respectively, in all cell types (n = 3; *P* = 0.001 or *P*<0.0001 for TNF-α alone versus each inhibitor; Figure S2A-C).

### The PPAR-γ agonist Inhibits TF mRNA Expression

**Figure 3:**
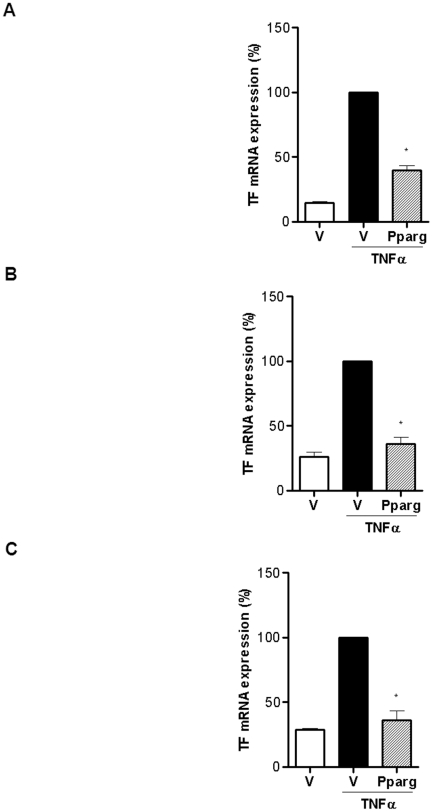
PPAR-γ agonist reduced TF mRNA expression in three cell types. (**A–C**) Real-time PCR demonstrated that TF mRNA levels increased after stimulation with TNF-α in HUVECs (A), THP-1 (B), and SMCs (C), which were suppressed by the PPAR-γ agonist. Data are presented as mean ± SEM. Values are given as percent of stimulation with TNF-α alone. *P<0.0001 vs TNF-α alone. All values are representative of 3 different experiments and are normalized to GAPDH. See also Figure S3.

We performed real-time PCR to quantitate the alterations of TF gene expression. TF mRNA level increased within 2 hours after stimulation with TNF-α. Pretreatment with the PPAR-γ agonist inhibited TF mRNA expression by 61±3% in HUVECs, by 64±5% in THP-1 and by 64±6% in SMCs (n = 3; *P*<0.0001 for TNF-α alone versus 25 µmol/L PPAR-γ agonist; Figure 3A-C, Figure S3A-C). These results suggest that the TF-lowering effects of the PPAR-γ agonist might be achieved through modification of transcription factors.

### The PPAR-γ agonist Reduces TF Promoter Activity

**Figure 4:**
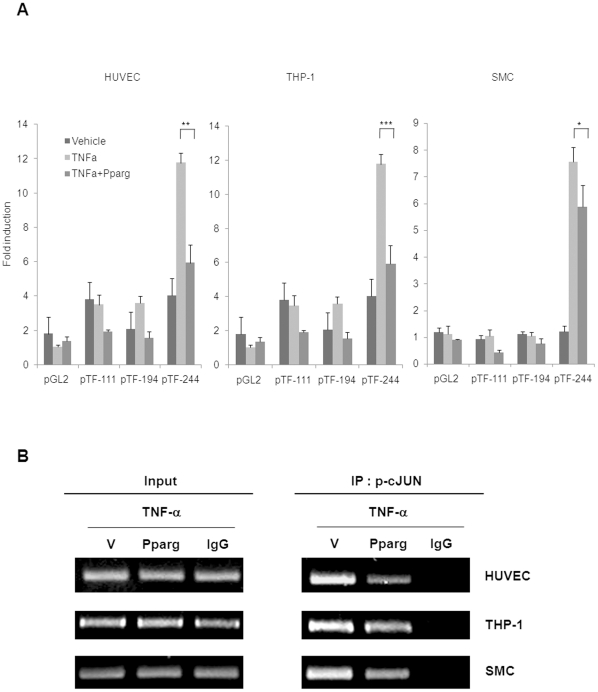
PPAR-γ agonist decreased TF promoter activity in three cell types. (**A**) The average fold induction of luciferase activity expressed by wild type or deletion mutant of TF promoter in response to the PPAR-γ agonist; three separate experiments were performed in triplicate (*P<0.05, **P<0.005, ***P<0.001). Data are presented as mean ± SEM. Luciferase assay demonstrated that AP-1 contributed to the inhibitory effect of the PPAR-γ agonist on TF expression in each cell type. (**B**) ChIP assay with AP-1 antibody verified that AP-1 was a critical transcription factor for TF-lowering effect of the PPAR-γ agonist in all cell types. See Figure S4 for the quantitative data.

To study the mechanism of the PPAR-γ agonist suppressing TF expression, we performed luciferase assay in three cell types. TNF-α significantly increased the promoter activity when plasmid pTF (-244) including wild-type TF promoter was transfected to HUVECs, whereas such activity was not observed when empty vector was transfected. Also, TNF-α-induced promoter activity was significantly decreased when plasmid with 5′-deletion between -244 and -194bp of the wild-type promoter was transfected, while it was not decreased anymore when even plasmid with further deletion to -111bp was transfected (Figure 4A). Because there is only activator protein-1 (AP-1) binding site between -244 and -194bp (Figure S4A), AP-1 might be a specific transcription factor for TF expression in HUVECs. We performed same experiments with this PPAR-γ agonist pretreated cells, and the inhibitory effect of the PPAR-γ agonist was the greatest in cells with wild-type vector transfected. It means that the inhibition of AP-1 binding to this promoter region was necessary for the PPAR-γ agonist to decrease TF expression (n = 3; *P*<0.005; Figure 4A). To verify these findings, we performed ChIP assay, which showed that the TF-lowering effect of the PPAR-γ agonist was mediated by inhibiting the activity of AP-1 binding to promoter region (n = 2; *P*<0.01; Figure 4B, Statistical graphs are shown in Figure S4B). In THP-1 and SMCs, similar patterns were observed (n = 3; *P*<0.001, *P*<0.05 respectively in each cell type; Figure 4A, n = 2; *P*<0.01, *P*<0.005 respectively in each cell type; Figure 4B, Statistical graphs are shown in Figure S4B), and so AP-1 could be a key target molecule for the PPAR-γ agonist to reduce TF level.

### The PPAR-γ agonist Reverses the Paclitaxel- or Rapamycin-induced Aggravation of TF Expression

**Figure 5:**
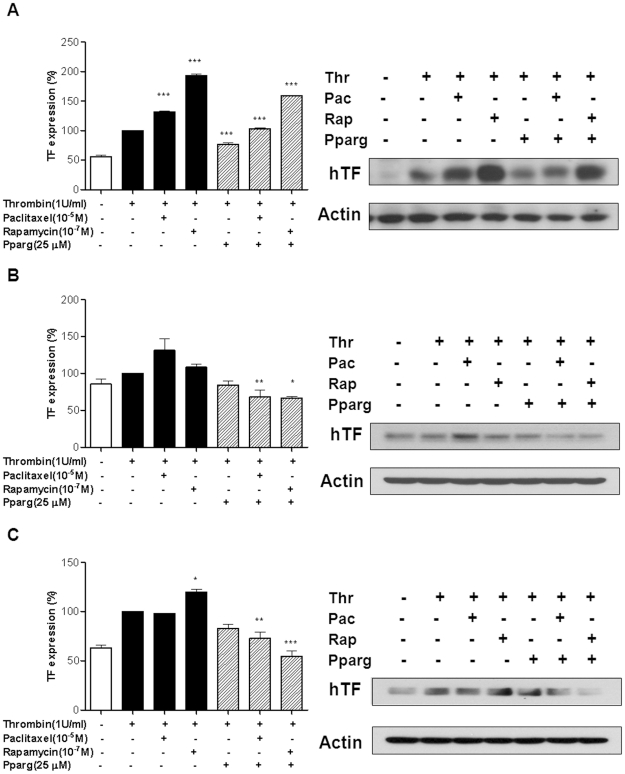
PPAR-γ agonist reversed the paclitaxel- or rapamycin-induced TF overexpression. (**A–C**) Paclitaxel and rapamycin aggravated thrombin-induced TF expression in HUVECs (A), THP-1 (B), and SMCs (C) (***P<0.0001 in HUVECs for paclitaxel or rapamycin vs thrombin alone; *P<0.05 in SMCs for rapamycin vs thrombin alone). The PPAR-γ agonist blunted the effect of paclitaxel and rapamycin on TF expression. Average values of 3 different experiments. Data are presented as mean ± SEM. Values are given as percent of stimulation with thrombin alone. *P<0.05, **P<0.01, ***P<0.0001 vs PPAR-γ agonist, PPAR-γ agonist plus paclitaxel, or PPAR-γ agonist plus rapamycin. Thr = thrombin; Pac = paclitaxel; Rap = rapamycin.

Paclitaxel and rapamycin are the most widely used drugs for drug-eluting stents and both are known to enhance TF expression [13]. We were curious whether the PPAR-γ agonist could prevent such unwanted thrombogenic effects of these drugs. Stimulation with thrombin increased TF expression of HUVECs, THP-1, and SMCs. Paclitaxel or rapamycin further aggravated thrombin-induced TF expression in these cells. The effects of paclitaxel or rapamycin were the most prominent in HUVECs (n = 3; *P*<0.0001 versus thrombin alone; Figure 5A). Weaker but same tendencies were observed in THP-1 and SMCs (Figure 5B-C). The PPAR-γ agonist decidedly attenuated the effects of paclitaxel or rapamycin on TF expression in all cell types (n = 3; *P*<0.0001 in HUVECs, *P* = 0.001, *P*<0.05 respectively in THP-1, *P* = 0.006, *P*<0.0001 respectively in SMCs versus thrombin plus paclitaxel or thrombin plus rapamycin; Figure 5A-C).

### The PPAR-γ agonist Does Not Reduce TFPI Expression in Three Different Cells

TFPI is a physiological inhibitor of TF. If the PPAR-γ agonist reduces TFPI expression more significantly, even if it decreases TF expression as shown above, it is hard to conclude that the PPAR-γ agonist can prevent thrombosis. Thus we investigated the effects of the PPAR-γ agonist on TFPI expression. TFPI expression was not affected by the PPAR-γ agonist in HUVECs and THP-1 (n = 3; NS for TNF-α alone versus 25 µmol/L PPAR-γ agonist; Figure S5A-B). However, in SMCs, the PPAR-γ agonist significantly increased TFPI expressions by 18±4% (n = 3; *P* = 0.005 for TNF-α alone versus 25 µmol/L PPAR-γ agonist; Figure S5C). These results suggest that the PPAR-γ agonist reduces TF expression without suppressing TFPI and that it has an anti-thrombogenic effect.

### The PPAR-γ agonist Reduces TF Expression and Induces TFPI Expression in vivo

**Figure 6:**
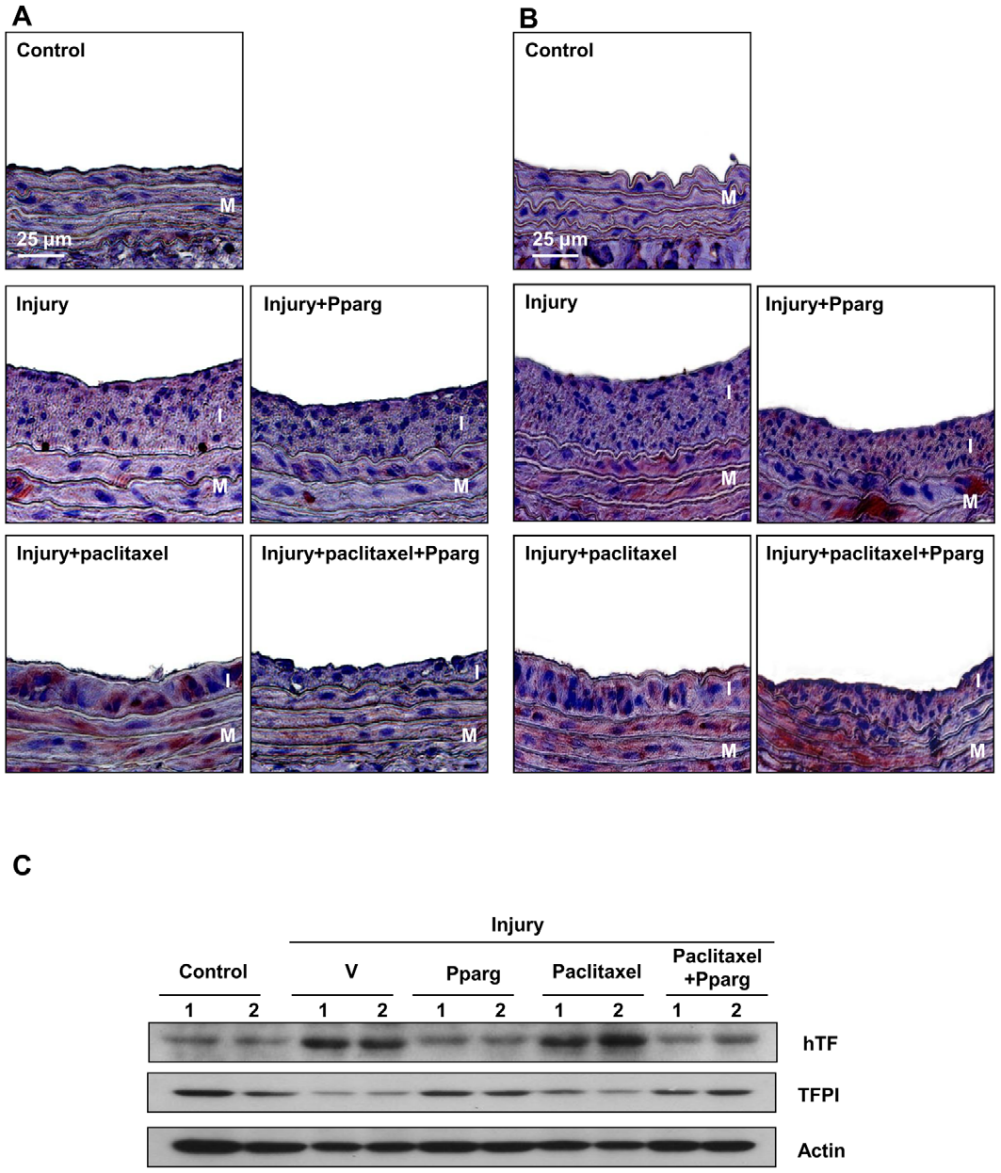
The changes of TF and TFPI expressions after PPAR-γ agonist treatment in vivo. (**A**) Representative images of IHC staining for TF (hematoxylin-eosin stain, magnification x400). Specimens prepared from the injured and uninjured rat carotid arteries were examined. Balloon injury increased TF expression of injured arteries. TF expression was less prominent after the PPAR-γ agonist treatment than that from balloon injury alone. Paclitaxel infusion enhanced TF expression, which was reversed by the PPAR-γ agonist. (**B**) Representative images of IHC staining for TFPI (hematoxylin-eosin stain, magnification x400). The PPAR-γ agonist increased TFPI expression with or without paclitaxel. Brownish-red indicates TF (A) or TFPI protein (B). The upper region indicates luminal side. I = intima; M = media. (**C**) Western blotting of carotid artery lysates confirmed these effects of the PPAR-γ agonist on TF and TFPI expressions. See Figure S6 and S7 for the low-power view of the rat carotid arteries, and for the quantitative data, respectively.

To clarify the effects of the PPAR-γ agonist on TF and TFPI expressions, we performed *in vivo* experiments using rat carotid injury model. Balloon injury increased not only neointimal formation as previously reported [15], but also TF expression in injured arteries (Figure 6A, C and Figure S6A). The PPAR-γ agonist reduced both neointimal thickness and TF expression induced by balloon injury (Figure 6A, C and Figure S6A). To mimic the condition of paclitaxel-eluting stent implantation, we continuously infused paclitaxel via mini-pump to rats. The addition of paclitaxel to balloon injury further enhanced TF expression, which was dramatically reversed by the PPAR-γ agonist (Figure 6A, C and Figure S6A). We also confirmed the effect of the PPAR-γ agonist on TFPI expression in this model; TFPI expression of injured arteries was further increased by the PPAR-γ agonist in the presence or absence of paclitaxel (Figure 6B-C, Figure S6B). Carotid artery lysates were also analyzed by western blotting, which demonstrated that the PPAR-γ agonist decreased TF expression by 70±5% while increased TFPI by 40±11% (n = 4; *P*<0.0001, *P*<0.05 respectively versus injury plus paclitaxel; Figure 6C, Statistical graphs are shown in Figure S7A-B).

## Discussion

Rosiglitazone has been restricted or suspended, since several studies have suggested that it might be associated with increased risk of myocardial infarction [5], [16]. However, the precise biological mechanisms responsible for the risk of myocardial infarction with rosiglitazone are uncertain. Quite recently, one paper demonstrated that the different distribution pattern of PPAR-γ and TF expressions in human coronary atherosclerotic plaques; this finding gives insight into the underlying mechanisms of the link between PPAR-γ agonists and myocardial infarction [17]. We hypothesized that PPAR-γ agonists might have a certain effect on TF expression, and this hypothesis is supported by our results. Here, we first suggest that the PPAR-γ agonist has anti-thrombogenic properties not due to its well-known anti-inflammatory [18] or anti-atherosclerotic effect [19] but due to its direct influence on TF expression. The PPAR-γ agonist reduced TNF-α-induced TF expression of ECs, monocytes, and SMCs. Furthermore, the PPAR-γ agonist increased TFPI expression, antithrombotic molecule, in SMCs. In vitro studies showed that the PPAR-γ agonist inhibited activation of MAPKs leading to inhibition of AP-1 binding to TF gene promoter in three cell types. The major type of affected MAPK was cell-type specific. Furthermore, we demonstrated that the PPAR-γ agonist reversed paclitaxel- or rapamycin-induced aggravation of thrombin-induced TF expression. To clarify these anti-thrombotic effects of the PPAR-γ agonist, we performed *in vivo* studies mimicking the condition of drug-eluting stents implantation and demonstrated, for the first time, that the PPAR-γ agonist reduced TF expression in the injured artery.

### TF as a Pivotal Molecule for Thrombus Generation

TF is a critical molecule that triggers pathologic thrombus formation [11]. TNF-α is a well-known cytokine that increases TF expression in ECs, monocytes, and VSMCs [11], [20]. We selected these three cell types for investigation because they were known as the most important cells participating in intravascular thrombosis [12]. VSMCs, which are the main sources of TF, constitutively express TF, and, thus, these cells promptly initiate coagulation in response to vessel wall damage [20]. In contrast, ECs and monocytes do not express TF under physiological conditions. Instead, TF expression can be rapidly induced by various stimuli in these cells [21]. This means that certain stimulants from one activated cell type can also enhance TF expression of the other cell types. According to our results, the PPAR-γ agonist has a remarkable TF-lowering effect on all these cell types, and it can be speculated that the net effect of the PPAR-γ agonist on TF expression might be greater than the mere sum of inhibitory effects on each cell type because the PPAR-γ agonist can attenuate the harmful communications among activated cells.

### Mechanism of the PPAR-γ agonist to Reduce TF Expression

Despite the diversity of stimuli augmenting TF expression, three MAPKs, including JNK, p38, and ERK, are involved in the most of the stimulant-induced TF expression [12]. These kinases activate TF promoter by enhancing activities of transcription factors such as AP-1, NF-κB, SP-1, and Egr-1 [22], [23]. Furthermore, one recent study showed that rosiglitazone could reduce ischemic injury in a non-diabetic mouse heart by modulating cardio-protective signaling pathways, including inhibition of JNK activation [24]. Taken together, we hypothesized that the PPAR-γ agonist would affect TF expression through MAPK pathway. We also assumed that cell type would determine the main MAPK influenced by the PPAR-γ agonist because we knew that each MAPK activation was not only stimulant-specific, but also cell type-specific [12]. Indeed, our result demonstrated that the PPAR-γ agonist reduced phosphorylation of JNK and p38 in HUVECs, p38 and ERK in THP-1, and JNK and p38 in SMCs. As a result of these MAPKs inhibitions, expression levels of TF mRNAs and corresponding proteins were decreased. We were also curious which transcription factor would be regulated by the PPAR-γ agonist and whether this regulation would be cell type-specific. According to 5′ deletion mutant promoter assay, we found that the major promoter sites for the response to the PPAR-γ agonist were located between -244 and -194, which is binding site for transcription factor AP-1. We further showed that AP-1 binding on this site was inhibited by the PPAR-γ agonist. Therefore, AP-1 was the key molecule regulated by this PPAR-γ agonist for reducing TF expression; these results are compatible with previous findings [25], [26]. However, we need to figure out whether the PPAR-γ agonist directly blocks the AP-1 activity or requires the cooperation of other factors. Here, we confirmed that the PPAR-γ agonist impaired TF expressions of three cell types and that this action was mediated by AP-1 suppression through cell type-specific inhibition of MAPK activity.

### Implication of the PPAR-γ agonist on the Risk of Stent Thrombosis

**Figure 7:**
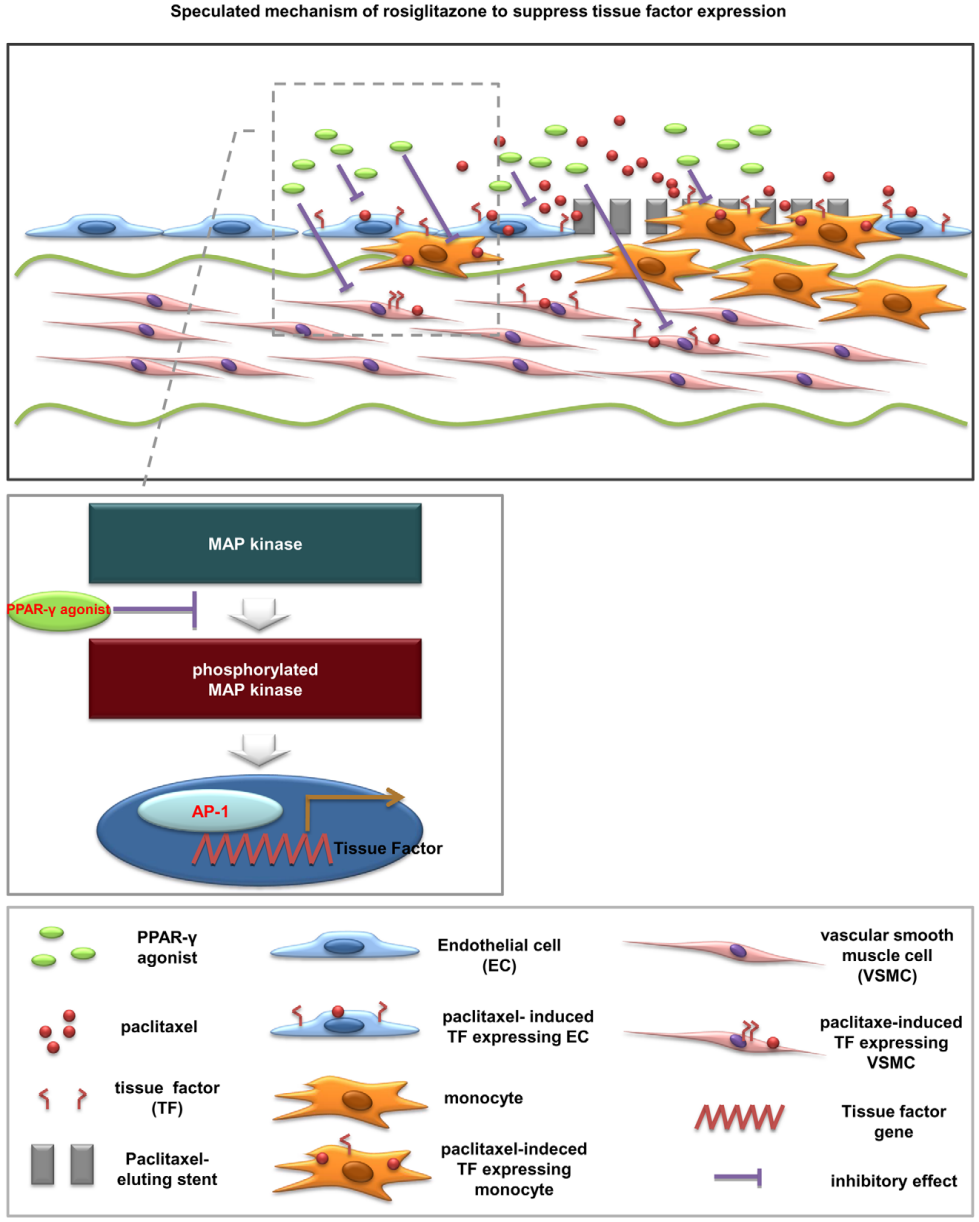
Speculated schematic illustration showing TF-lowering effect of PPAR-γ agonist in three cell types. In addition to acute coronary syndrome itself and endothelial denudation after stent implantation, paclitaxel induced thrombosis-prone condition by enhancing TF expression in ECs, monocytes and SMCs. Our study suggested that the PPAR-γ agonist suppressed TF expression in these cell types and this action is mediated by the inhibition of MAP kinase and AP-1 activity.

Drug-eluting stents is a mainstay of treatment for ischemic heart disease. However, these devices possess the risk of stent thrombosis, a fatal complication caused by thrombus formation inside stents. Two main types of drug-eluting stents, paclitaxel- or silorimus-eluting stents, unintentionally retard endothelial regeneration and accordingly heighten the risk of late and very late stent thrombosis [27], [28]. _ENREF_29Furthermore, paclitaxel and rapamycin are known to enhance TF expression [13], [14]. Our results showed that the PPAR-γ agonist decreased paclitaxel- or rapamycin-induced TF over-expressions in ECs, monocytes, and SMCs. VSMCs are exposed to the bloodstream after stent implantation, and, therefore, TF in VSMCs also comes into contact with coagulation factors. The inflammatory milieu during acute coronary syndrome, procedure-related vascular injuries or eluted drugs from stents can induce TF expression in residual ECs and migrated monocytes [29]. In this regard, it can be speculated that increased risk of stent thrombosis after drug-eluting stents implantation due in part to enhanced TF expression within the vasculatures. As mentioned above, stimulatory interaction between cells can aggravates TF expression synergistically. Thus this PPAR-γ agonist might be an attractive option that can reduce TF expressions in all cell types as shown in our study (Figure 7).

### TFPI as Counterpart of TF

The extent of TF protein induction does not always correlate with TF activity [30]. One reason for this discrepancy is the concurrent secretion of TFPI, the key endogenous inhibitor of TF. Therefore, we evaluated the change of TFPI after the PPAR-γ agonist treatment in the same way as TF. TFPI is mainly synthesized and released by ECs under physiological condition [31], and it is subsequently acknowledged that monocytes and VSMCs also express TFPI [32]. Our data demonstrated that the PPAR-γ agonist did not change TFPI expression significantly in HUVECs and THP-1. Moreover, the PPAR-γ agonist increased TFPI expression in VSMCs. According to our data, it can be speculated that this PPAR-γ agonist can reduce thrombogenecity of atherosclerotic plaque by suppressing TF expression without decreasing TFPI expression in vasculatures.

### Appropriateness of Rat Balloon Injury Model

To confirm the effect of the PPAR-γ agonist on TF expression in the setting of drug-eluting stents implantation, we performed *in vivo* experiments using rat balloon injury model. Previous studies showed that the balloon injury on rat carotid vessels increased TF expression [33]. Drug-eluting stents implantation induces TF expression by direct exposure of VSMCs to circulating blood due to denuded endothelium [34] and by the effect of drug released from stent. To mimic this condition, we combined paclitaxel treatment with balloon injury. Previous studies showed that rats treated with paclitaxel by IP injection at 2 mg/kg/day successfully decreased neointima formation [35]. We demonstrated similar effect with paclitaxel by continuous intravascular infusion at 0.3 mg/kg/day. We infused paclitaxel via mini-pump instead of IP injection for several reasons. First, we attempted to make intravascular infusion condition similar to that of drug-eluting stents implantation in the aspect of continuous delivery of the drug. Secondly, our technique was more efficient at avoiding the error due to misplacement of IP injection, resulting in decreased drug delivery and underestimation of paclitaxel effect. Our data showed that paclitaxel reduced neointima formation as expected, but it increased TF expression in vasculatures. This paclitaxel-induced overexpression of TF was reversed by the PPAR-γ agonist. However, its physiological inhibitor, TFPI, was increased by the PPAR-γ agonist. The effect of the PPAR-γ agonist on neointima formation was not evaluated in this study, but we recently suggested a novel mechanism that this PPAR-γ agonist attenuated neointima formation through glycogen synthase kinase-3β (GSK-3β) activation [36]. Because GSK-3β is known as a negative regulator of platelet function [37],_ENREF_38 this would be another mechanism of anti-thrombotic action of the PPAR-γ agonist. Taken together, our finding suggests the possibility of its usefulness in the prevention of stent thrombosis. Furthermore, our data may have another clinical relevance; the considerable efficacy of short-term treatment (9 days) with rosiglitazone can give a chance to avoid potential adverse effects of this drug by ‘limiting the duration of treatment to periprocedural phase of stent implantation’. However, it is more plausible to evaluate the effects of rosiglitazone on TF and TFPI expressions in normal vessels without balloon injury as well as the effects of a prolonged exposure of the drug prior to balloon injury, since these models are more relevant to the clinical situation in which diabetic patients take the drug for a long time before having myocardial infarction. In this context, we also tried to analyze the effects of rosiglitazone on TF and TFPI expressions in corresponding contralateral uninjured carotid arteries. Although the effects of the PPAR-γ agonist on TF and TFPI expressions in the uninjured arteries were not dramatic compared to those in the injured arteries, the drug could reverse paclitaxel-induced TF expression without reducing TFPI expression (Figure S8A-B). Accordingly, it can be speculated that rosiglitazone may have a protective, or, at least, no harmful effect on thrombogenesis in the normal vessels as well as the vessels with stent implantation. Further experiments using diabetes animal model with long-term rosiglitazone administration would be the next step in order to validate this effect and to provide a biological answer to the concerns over cardiovascular safety of rosiglitazone. More importantly, thrombosis model are needed to confirm a hitherto unrecognized role of PPAR-γ agonists: a potential treatment to reduce the risk of stent thrombosis. Moreover, future clinical researches for patients with drug-eluting stents implantation can build on these observations.

In conclusion, our results provide the evidence that the PPAR-γ agonist reduces TF expression by inhibition of MAPK pathway and suppression of downstream transcription factor, AP-1 binding activity. The PPAR-γ agonist also reverses paclitaxel-induced aggravation of TF expression. Thus, our study suggests a possible speculation to the link between myocardial infarction and rosiglitazone use, which might be against the existing assumptions. Furthermore, although further clinical studies are required, it can be speculated that the benefits of this agent might outweigh its risks in a certain group of patients with drug-eluting stents implanted.

## Materials and Methods

### Ethics Statement

The investigation using human umbilical arterial SMCs conforms with the principles outlined in the Declaration of Helsinki and was approved by the Clinical Research Institute in Seoul National University Hospital (IRB No. H-0905-031-281). Written informed consent was received from all participants. All animal experiments were performed after receiving approval from the Institutional Animal Care and Use Committee (IACUC), Clinical Research Institute, Seoul National University Hospital (IACUC No. 08-0133). The experimental protocol was designed in accordance with the Guide for Experimental Animal Research issued by the Laboratory for Experimental Animal Research, Clinical Research Institute, Seoul National University Hospital. And the investigation conforms to the Guide for the Care and Use of Laboratory Animals published by the United States National Institutes of Health (NIH Publication No. 85-23, revised 1996).

### Cells and Cell Cultures

HUVECs, characterized by Clonetics, were grown in endothelial growth medium (Clonetics, Walkersville, MD, USA) supplemented with endothelial basal medium, 5% fetal bovine serum (FBS), human epithelial growth factor, gentamicin & amphotericin (GA-1000) and bovine brain extract. HUVECs between passages 3 and 6 were used.

THP-1, obtained from Korean Cell Line Bank, was cultured in RPMI-1640 medium (WelGENE Inc., Daegu, Korea) supplemented with 10% FBS.

Human umbilical arteries were obtained from the umbilical cord. SMCs were isolated by enzymatic dispersion as previously described [38]. Umbilical arteries were cut into small pieces, placed in Dulbecco's modified Eagle's medium (DMEM) (Gibco, California, USA) containing 0.1% bovine serum albumin (Sigma Chemical Co., St. Louis, MO, USA) and collagenase type II (Gibco, California, USA). Then, cells were cultured in low glucose DMEM containing 10% FBS with 1% antibiotic/antimycotic solution (Gibco, Caifornia, USA).

All these cells are grown at 37°C in a humidified atmosphere containing 5% CO_2_. Initial pH in each media solution was adjusted to 7.4 with sodium hydroxide or hydrochloric acid. Cells have been authenticated by morphological observation and identification of cell-specific expression markers within one month before experiment.

### Western Blot Analysis

Cells were incubated in serum deprived media for 24 hours and then stimulated with 5 ng/mL TNF-α (R&D systems, Minneapolis, MN, USA) for 5 hours; different concentration of rosiglitazone (0, 5, 10, and 25 µmol/L, GlaxoSmithKline, UK) was treated for 1 hour before stimulation. We selected these doses of rosiglitazone because, according to previous studies, they were shown to optimally exhibit desired effects such as insulin sensitization in the in vitro experiments [39], [40]. Furthermore, these doses seem to be far from toxic doses in terms of EC 50 values, as suggested from previous data [41]. Taken together, we speculated the doses of the PPAR-gamma agonist used in our in vitro experiment might be comparable with the clinical doses. Western blot analysis was performed as described previously [42]. Protein was separated by SDS-polyacrylamide electrophoresis gel and transferred to a polyvinylidene difluoride membrane (Millipore). The membrane was blocked with T-TBS (1xTBS, 0.05% Tween-20) containing 3% skim milk and incubated with primary antibody overnight at 4°C. After 3 washes with T-TBS, the membrane was incubated with secondary antibody for 1 hour at room temperature. ECL or ECL-PLUS (Amersham) was used for detection. To reprobe the membrane, it was treated with Restore Western Blot stripping buffer (Pierce). Primary antibodies used in this study were as follows: anti-human TF (American Diagnostica Inc., Stamford, CT, USA), anti-human TF pathway inhibitor (TFPI) (Santa Cruz, CA, USA), anti-phospho-JNK, anti-phospho-p38, anti-phospho-ERK2/1, anti-JNK, anti-p38, anti-ERK2/1 (Cell signaling, Berkely, MA, USA) and anti-actin (Santa Cruz, CA, USA). In experiments using chemical blockers, SP600125, SB203580, and PD98059 (Calbiochem, San Diego, CA, USA) were used (1, 5, and 3 µmol/L respectively).

### Real-time Polymerase Chain Reaction (PCR)

Total RNA was extracted using Trizol Reagent (Invitrogen, California, USA) according to the manufacturer's instructions. One microgram of total RNA was used for reverse transcription. Real-time PCR was performed using an ABI prism 7500 (Applied Biosystems, California, USA) with SYBR Green master mix (Roche Applied Science, Mannheim, Germany). The cycling conditions consisted of 50°C for 2 min, 95°C for 10 min, and 95°C 15 s, 60°C for 1 min for 40 cycles and the melting point was determined and dissociation curves were obtained to assure the specificity of the reaction. The primers were as follows: forward 5′-agagttcacaccttacctgga-3′, reverse 5′-agttttctcctttatccacat-3′ for human TF and forward 5′-gagtcaacggatttggtcgt-3′, reverse 5′-gacaagcttcccgttctcag-3′ for GAPDH. Reverse-Transcriptase Polymerase Chain Reaction (RT-PCR) was also performed with the paired primers as follows: forward 5′-agagttcacaccttacctgga-3′, reverse 5′-agttttctcctttatccacat-3′ for human TF and forward 5′-cgtggaaggactcatgac-3′, reverse 5′-caaattcgttgtcataccag-3′ for GAPDH.

### Luciferase Promoter Assay

The wild type (pTF(-244)LUC) and deletion mutants (pTF(-194)LUC, pTF(-111)LUC) of human TF promoter have been described previously [43]. Briefly, DNA fragments from the TF promoter were inserted into the multicloning site of pl9luc. Mutated plasmids were made by site-directed mutagenesis using oligonucleotides which contain the desired mutations. These plasmids were transfected to HUVECs, THP-1 and SMCs, and these cells were stimulated with TNF-α. To verify the effect of the PPAR-γ agonist, each cell type was pretreated with this PPAR-γ agonist, and then was stimulated with TNF-α. Luciferase activities were measured using the Promega luciferase assay system and were normalized with β–galactosidase activities to correct for transfection efficiency.

### Chromatin Immunoprecipitation (ChIP)

ChIP assay was performed using a commercially available kit (Upstate Biotechnology, Lake Placid, NY, USA) following the manufacturer's instructions. Antibodies for phospho-c-Jun (Cell signaling, Berkely, MA, USA) were used for immunoprecipitation of the DNA fragments. The precipitated DNA fragments were analyzed by PCR with primers specific for the human TF promoter (forward; 5′-ccctccctttcctgccatag-3′, reverse; 5′-tcctcccggtaggaaactcc-3′).

### Immunohistochemical (IHC) Staining

IHC was performed as previously described [44]. Briefly, the paraffin-embedded samples were sectioned and treated with protease K for 4 minutes. Endogenous peroxidase was quenched with methanol/peroxidase solution. The specimens were treated with 50 mmol/L Tris HCl (pH 7.6) containing 0.15 mol/L NaCl and 0.1% Tween 20 for 5 minutes, followed by incubation with primary antibodies. The specimens were then processed by incubation with 1∶50 diluted 3,39-diaminobenzidine tetrahydrochloride substrate solution (DAKO) and counterstained with Mayer hematoxylin (DAKO). The primary antibodies used were anti-TF (American Diagnostica Inc., Stamford, CT, USA), and anti-TFPI (Santa Cruz, CA, USA).

### Rat Carotid Artery Balloon Injury Model

Rat balloon injury model has been described previously [36]. Forty male *Sprague-Dawley* rats, 9 weeks old, weighing around 200 g (Daehan Biolink Co., Chungcheongbuk-Do, Korea) were grouped into four groups (n = 10/group): balloon injury, balloon injury with the PPAR-γ agonist injection, balloon injury with paclitaxel infusion, and balloon injury with the PPAR-γ agonist injection and paclitaxel infusion. The PPAR-γ agonist (10 mg/kg) and 10% dimethyl sulfoxide (DMSO) as a solvent or 10% DMSO alone was administered intraperitoneally (IP) to rats for 2 days until the balloon injury and for 7 days after the injury. We selected this dose of rosiglitazone according to previous paper, which suggested that the therapeutic index for this drug would be >3 and ≤10 mg/kg in the animal model using rats [45]. Therefore, it can be speculated that the dose of 10 mg/kg of rosiglitazone may be appropriate to evaluate the TF-lowering effect of the drug in our in vivo experiment. All rats were fed with standard pellet feed and given water *ad libitum*. Animals were anaesthetised with ketamine hydrochloride and xylazine (50 mg/kg, 7 mg/kg IP respectively, Yuhan Co., Bayer Korea, Seoul, Korea) and all efforts were made to ameliorate suffering of animals. Noxious stimuli were applied to a limb occasionally throughout the experiments while monitoring changes in end-tidal carbon dioxide, heart rate, blood pressure, and cardiac rhythm in order to ascertain adequacy of anaesthesia. The left carotid artery was exposed, and a 2F Fogarty balloon embolectomy catheter (Baxter, Irvine, CA, USA) was inserted via an external carotid arteriotomy incision. The catheter was advanced to the common carotid artery, inflated with 0.2 mL of saline, and withdrawn 3 times with rotation. After balloon injury, 2F Fogarty balloon embolectomy catheter was removed, and dwelling catheter was inserted to the arteriotomy site. Then, the external carotid artery was ligated. Paclitaxel was continuously administered for 7 days by osmotic mini-pump (Alzet®, Durect, Cupertino, CA, USA) at 0.3 mg/kg/day. The right internal jugular vein was cannulated using a commercially available rat jugular catheter (Durect, Cupertino, CA, USA) which was connected to mini-pump that was implanted subcutaneously as previous described [46]. In the group without paclitaxel infusion, the sham operation was done in the same manner except that no mini-pump or jugular catheter was inserted. One week after the injury, rats were killed by pentobarbital overdose and perfused with 10% formaldehyde. Carotid arteries were removed and placed in the same fixative. Tissues were embedded in paraffin, and 4 to 5 sections (4 µm) were cut at multiple levels.

### Statistical Analysis

Data are presented as mean ± standard error of the mean (SEM). Statistical analysis was performed by Student *t*-test or ANOVA as appropriate. SPSS version 18.0 was used for analysis. *P*-value of <0.05 was considered statistically significant.

## Supporting Information

Figure S1 **Cell type-specific inhibition of MAPK phosphorylation.** (**A–C**) The overexpression of TF was mediated by the increased phosphorylation of MAPK, which was blocked by the PPAR-γ agonist. The main MAPK varied depending on each cell type; HUVECs were dependent on JNK and p38 (A); THP-1 cells were dependent on p38 and ERK (B); SMCs were dependent on JNK and p38 (C). Average values of 3 different experiments. Data are presented as mean ± SEM. *P<0.05, **P<0.01, ***P<0.0001 vs TNF-α alone.
(TIF)

Figure S2 **The effects of MAPK inhibitors on TF expression.** (**A–C**) In order to verify the involvement of MAPK in TNF-α-induced TF overexpression under our experimental conditions, the effects of MAPK inhibitors on TF expression was examined in HUVECs (A), THP-1 (B) and SMCs (C). SP600125 (10^−6^ mol/L), SB203580 (10^−5^ mol/L), and PD98059 (3×10^−6^mol/L), specific inhibitors of JNK, p38, and ERK, respectively, impaired TF induction after TNF-α stimulation in all cell types. Average values of 3 different experiments. Data are presented as mean ± SEM. Values are given as percent of stimulation with TNF-α alone. *P<0.05, **P = 0.001, ***P<0.0001 vs TNF-α alone.
(TIF)

Figure S3 **Reduction in TF mRNA expression by PPAR-γ agonist in three cell types.** (**A–C**) RT-PCR demonstrated that TF mRNA levels increased after stimulation with TNF-α in HUVECs (A), THP-1 (B), and SMCs (C), which were suppressed by the PPAR-γ agonist. Values are given as percent of stimulation with TNF-α alone. *P<0.0001 vs TNF-α alone. All values are representative of 3 different experiments and are normalized to GAPDH. Data are presented as mean ± SEM.
(TIF)

Figure S4 **PPAR-γ agonist decreased TF promoter activity in three cell types.** (**A**) Constructs of transfected plasmids. Note that there is only AP-1 binding site between -244 and -194bp. (**B**) ChIP assay with AP-1 antibody verified that AP-1 was a critical transcription factor for TF-lowering effect of the PPAR-γ agonist in all cell types. Average values of 2 different experiments. Data are presented as mean ± SEM. Values are given as percent of stimulation with TNF-α alone. *P<0.01, **P<0.005.
(TIF)

Figure S5 **The effects of PPAR-γ agonist on TFPI expression in three different cells.** (**A–B**) The PPAR-γ agonist did not affect TFPI expression in HUVECs (A) and THP-1 (B). *P* = NS vs TNF-α alone. (**C**) The PPAR-γ agonist increased TFPI expression in SMCs. Average values of 3 different experiments. Data are presented as mean ± SEM. Values are given as percent of stimulation with TNF-α alone. *P = 0.005, **P = 0.001 vs TNF-α alone.
(TIF)

Figure S6 **Low-power view of the rat carotid arteries with TF or TFPI expression.** (**A–B**) Representative images of IHC staining for TF or TFPI (hematoxylin-eosin stain, OLYMPUS IX71, magnification x40, colors corrected after acquisition with Adobe Photoshop). TF levels showed a tendency to decrease with the PPAR-γ agonist treatment in the presence or absence of paclitaxel (A). An opposite tendency was observed regarding TFPI expression in the same specimen (B). Brownish-red indicates TF (left panel) or TFPI protein (right panel).
(TIF)

Figure S7 **The changes of TF and TFPI expressions after PPAR-γ agonist treatment in vivo.** (**A–B**) Bar graphs showed quantitative data for TF (A) and TFPI expressions (B) that are normalized to actin; Separate experiments were performed with 4 different rats per group. Data are presented as mean ± SEM. Values are given as percent of stimulation with balloon injury alone. *P<0.05, **P<0.01***P<0.0001 vs PPAR-γ agonist, or PPAR-γ agonist plus paclitaxel.
(TIF)

Figure S8 **The effects of PPAR-γ agonist on TF and TFPI expressions in corresponding contralateral uninjured carotid arteries.** (**A–B**) Representative images of IHC staining for TF or TFPI (hematoxylin-eosin stain, magnification x40). Paclitaxel without balloon injury slightly increased TF expression, which was reversed by the PPAR-γ agonist (A). With respect to TFPI expression, a minimal increase was observed in the uninjured artery treated with paclitaxel plus rosiglitazone, as compared to that treated with paclitaxel alone (B). Brownish-red indicates TF (upper panel) or TFPI protein (lower panel).
(TIF)
