# Supplementary figures and images for: Peroxisome Proliferator-Activated Receptor-Gamma Agonists Suppress Tissue Factor Overexpression in Rat Balloon Injury Model with Paclitaxel Infusion
Source: PLoS One. 2011 Nov 29;6(11):e28327. doi: 10.1371/journal.pone.0028327 (PMC3226685; doi:10.1371/journal.pone.0028327)

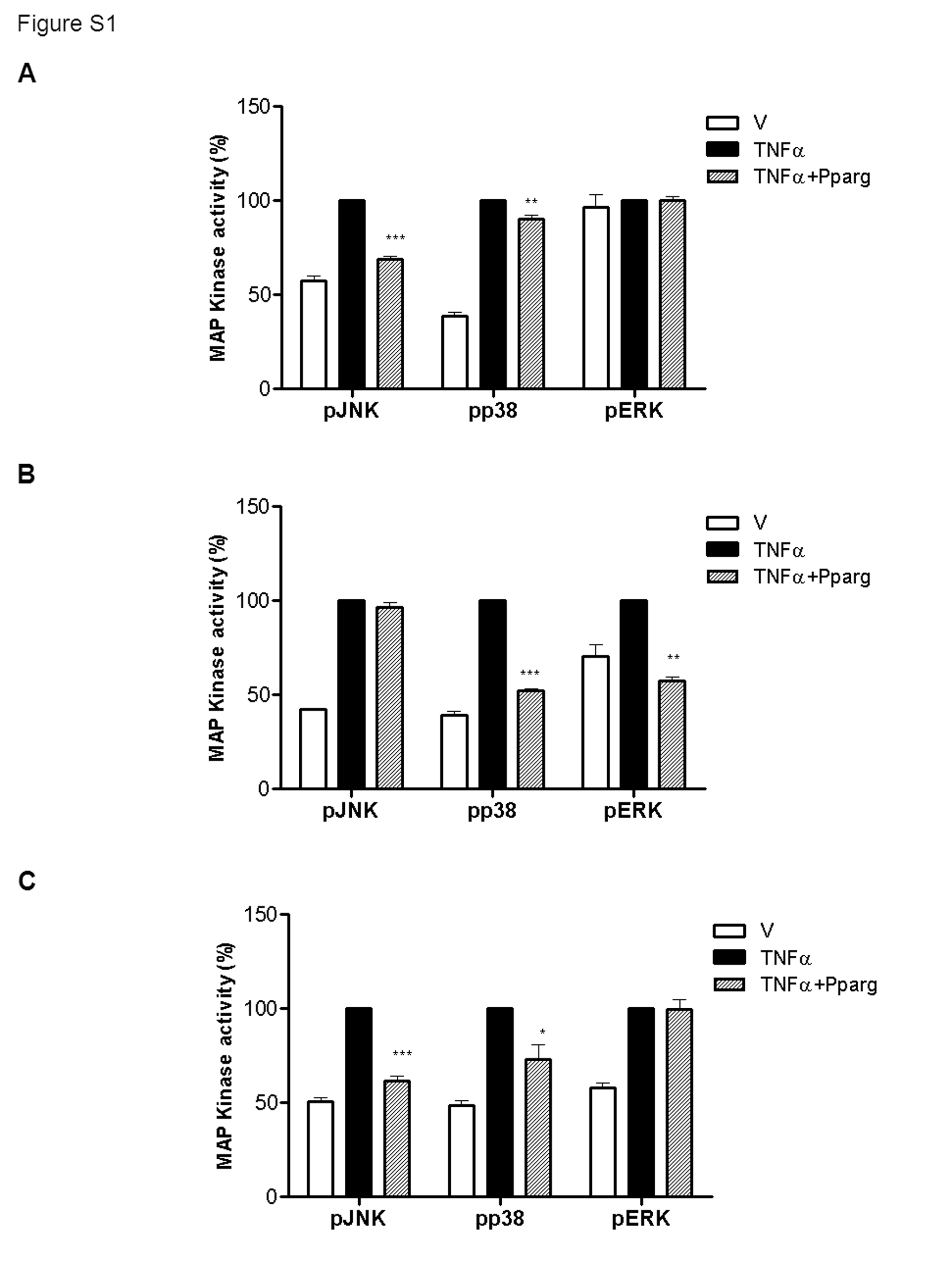

Supplement: Figure S1 — Cell type-specific inhibition of MAPK phosphorylation. (A–C) The overexpression of TF was mediated by the increased phosphorylation of MAPK, which was blocked by the PPAR-γ agonist. The main MAPK varied depending on each cell type; HUVECs were dependent on JNK and p38 (A); THP-1 cells were dependent on p38 and ERK (B); SMCs were dependent on JNK and p38 (C). Average values of 3 different experiments. Data are presented as mean ± SEM. *P<0.05, **P<0.01, ***P<0.0001 vs TNF-α alone. (TIF) [file pone.0028327.s001.tif]

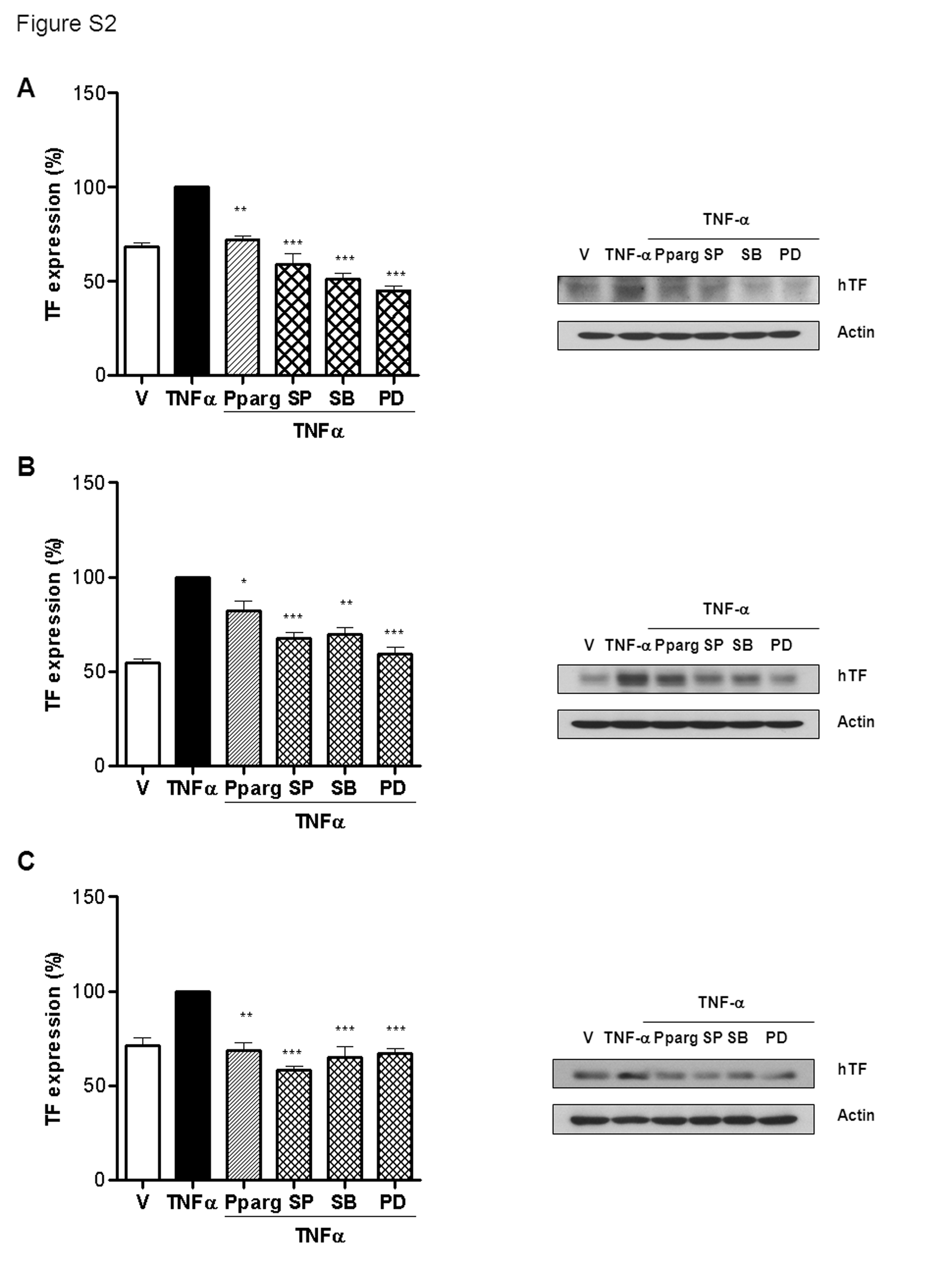

Supplement: Figure S2 — The effects of MAPK inhibitors on TF expression. (A–C) In order to verify the involvement of MAPK in TNF-α-induced TF overexpression under our experimental conditions, the effects of MAPK inhibitors on TF expression was examined in HUVECs (A), THP-1 (B) and SMCs (C). SP600125 (10−6 mol/L), SB203580 (10−5 mol/L), and PD98059 (3×10^−6^mol/L), specific inhibitors of JNK, p38, and ERK, respectively, impaired TF induction after TNF-α stimulation in all cell types. Average values of 3 different experiments. Data are presented as mean ± SEM. Values are given as percent of stimulation with TNF-α alone. *P<0.05, **P = 0.001, ***P<0.0001 vs TNF-α alone. (TIF) [file pone.0028327.s002.tif]

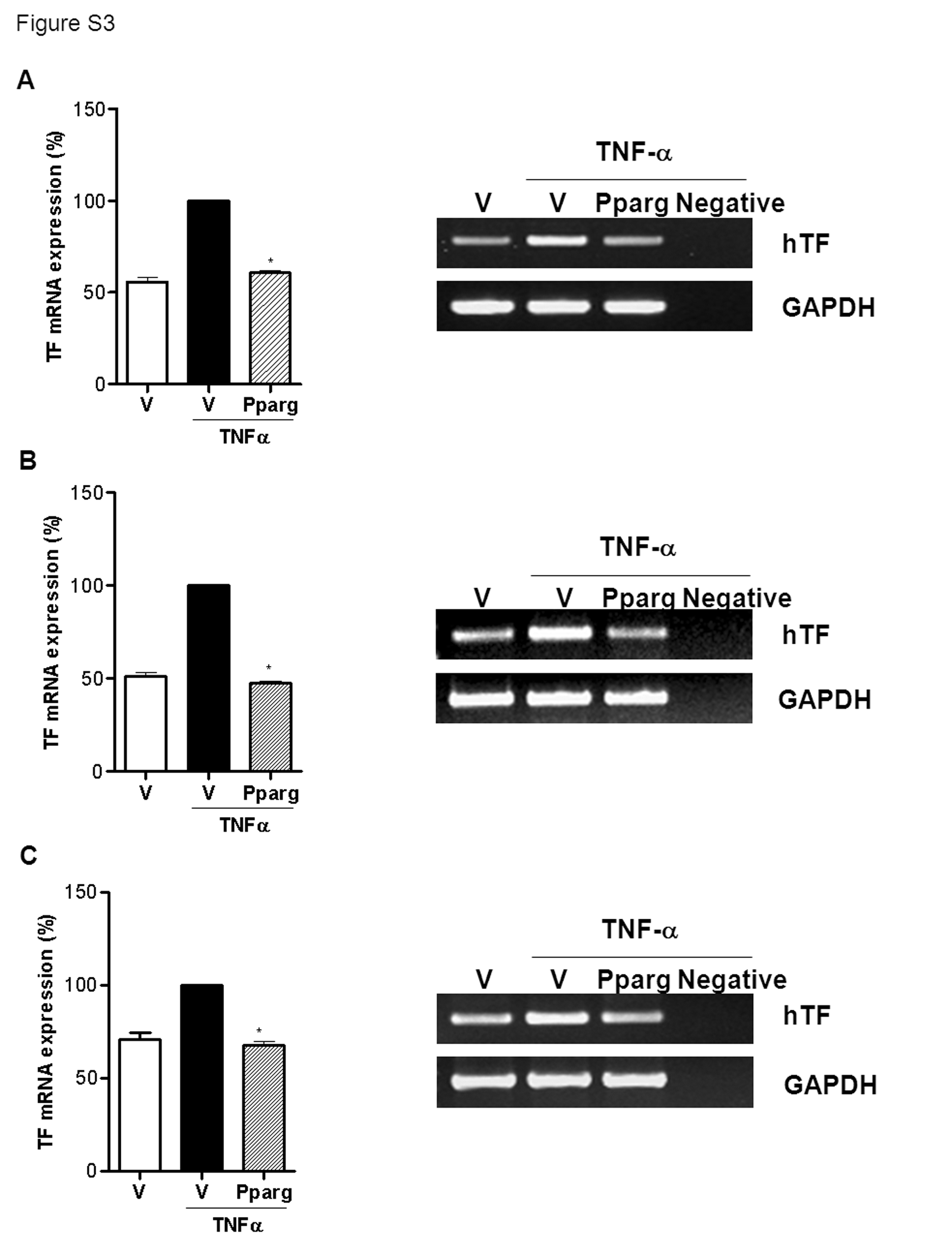

Supplement: Figure S3 — Reduction in TF mRNA expression by PPAR-γ agonist in three cell types. (A–C) RT-PCR demonstrated that TF mRNA levels increased after stimulation with TNF-α in HUVECs (A), THP-1 (B), and SMCs (C), which were suppressed by the PPAR-γ agonist. Values are given as percent of stimulation with TNF-α alone. *P<0.0001 vs TNF-α alone. All values are representative of 3 different experiments and are normalized to GAPDH. Data are presented as mean ± SEM. (TIF) [file pone.0028327.s003.tif]

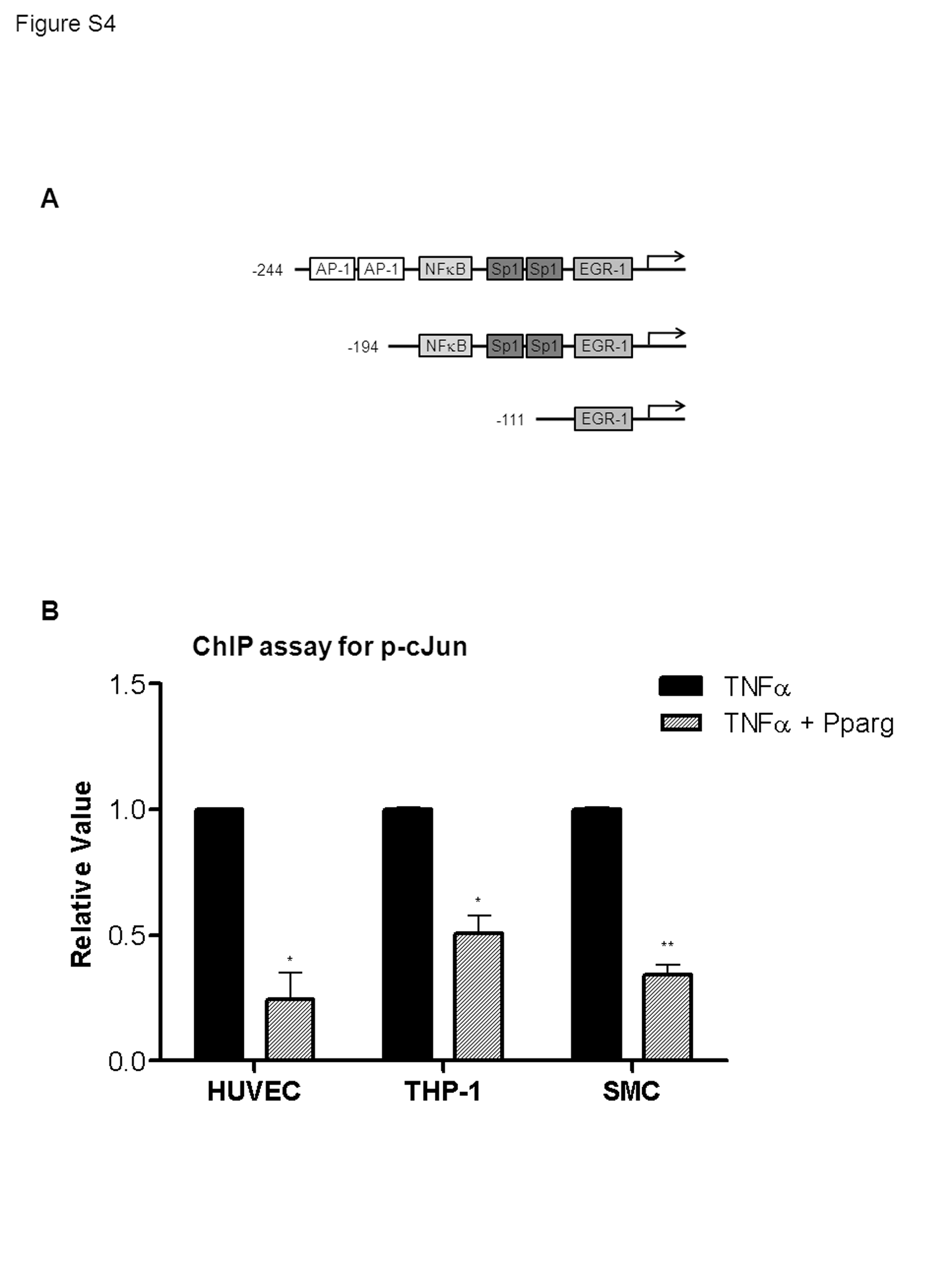

Supplement: Figure S4 — PPAR-γ agonist decreased TF promoter activity in three cell types. (A) Constructs of transfected plasmids. Note that there is only AP-1 binding site between -244 and -194bp. (B) ChIP assay with AP-1 antibody verified that AP-1 was a critical transcription factor for TF-lowering effect of the PPAR-γ agonist in all cell types. Average values of 2 different experiments. Data are presented as mean ± SEM. Values are given as percent of stimulation with TNF-α alone. *P<0.01, **P<0.005. (TIF) [file pone.0028327.s004.tif]

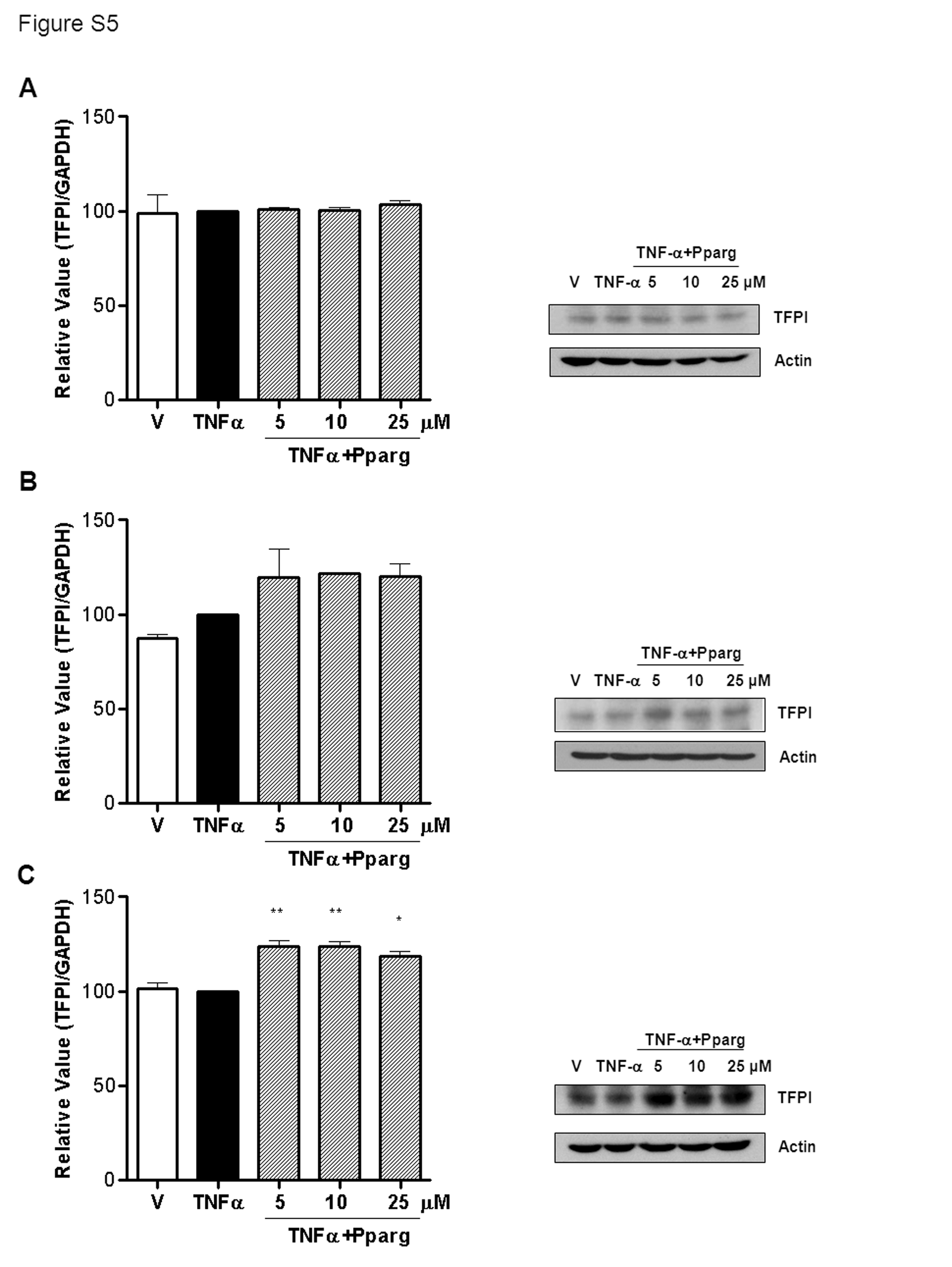

Supplement: Figure S5 — The effects of PPAR-γ agonist on TFPI expression in three different cells. (A–B) The PPAR-γ agonist did not affect TFPI expression in HUVECs (A) and THP-1 (B). P = NS vs TNF-α alone. (C) The PPAR-γ agonist increased TFPI expression in SMCs. Average values of 3 different experiments. Data are presented as mean ± SEM. Values are given as percent of stimulation with TNF-α alone. *P = 0.005, **P = 0.001 vs TNF-α alone. (TIF) [file pone.0028327.s005.tif]

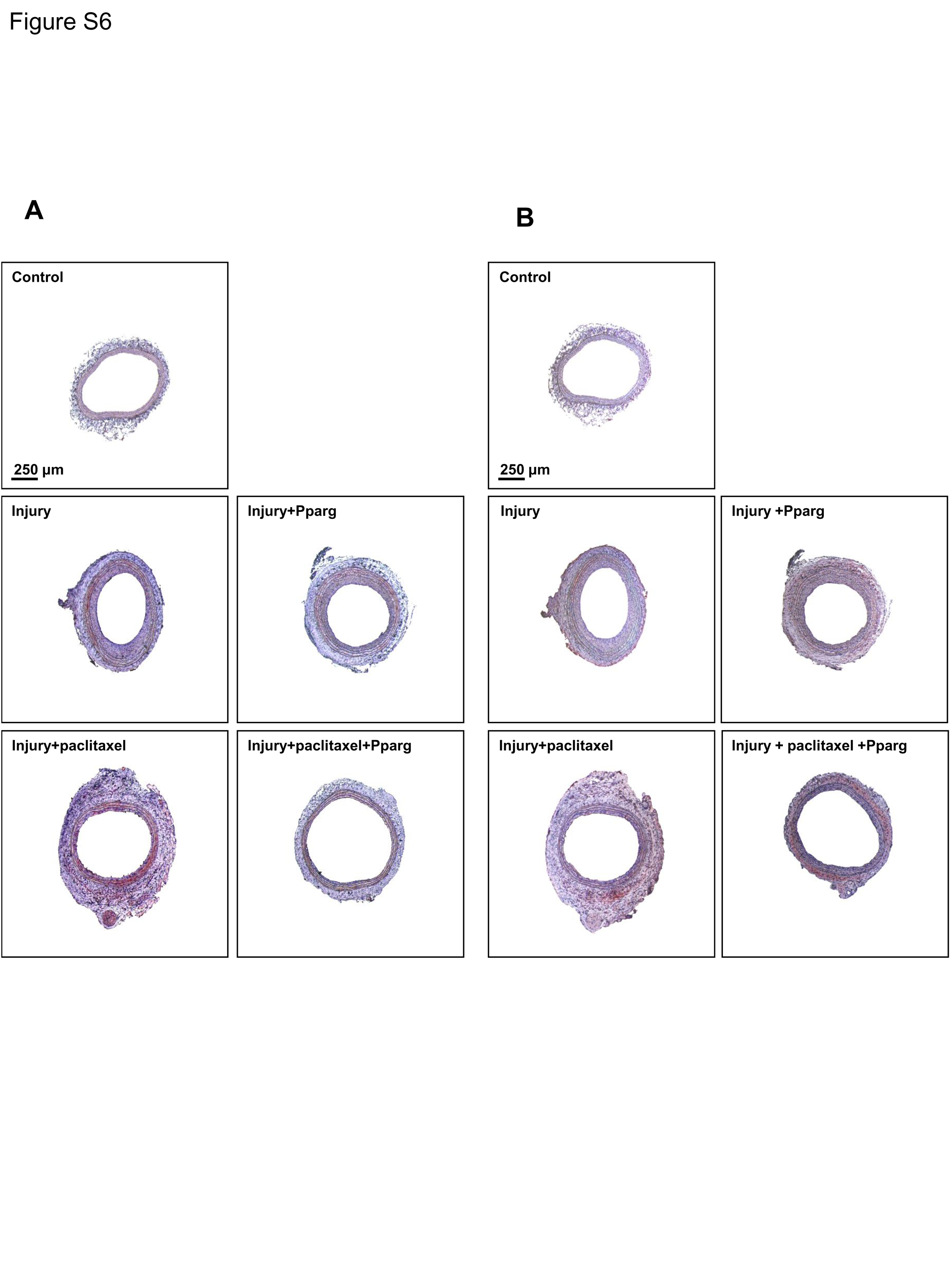

Supplement: Figure S6 — Low-power view of the rat carotid arteries with TF or TFPI expression. (A–B) Representative images of IHC staining for TF or TFPI (hematoxylin-eosin stain, OLYMPUS IX71, magnification x40, colors corrected after acquisition with Adobe Photoshop). TF levels showed a tendency to decrease with the PPAR-γ agonist treatment in the presence or absence of paclitaxel (A). An opposite tendency was observed regarding TFPI expression in the same specimen (B). Brownish-red indicates TF (left panel) or TFPI protein (right panel). (TIF) [file pone.0028327.s006.tif]

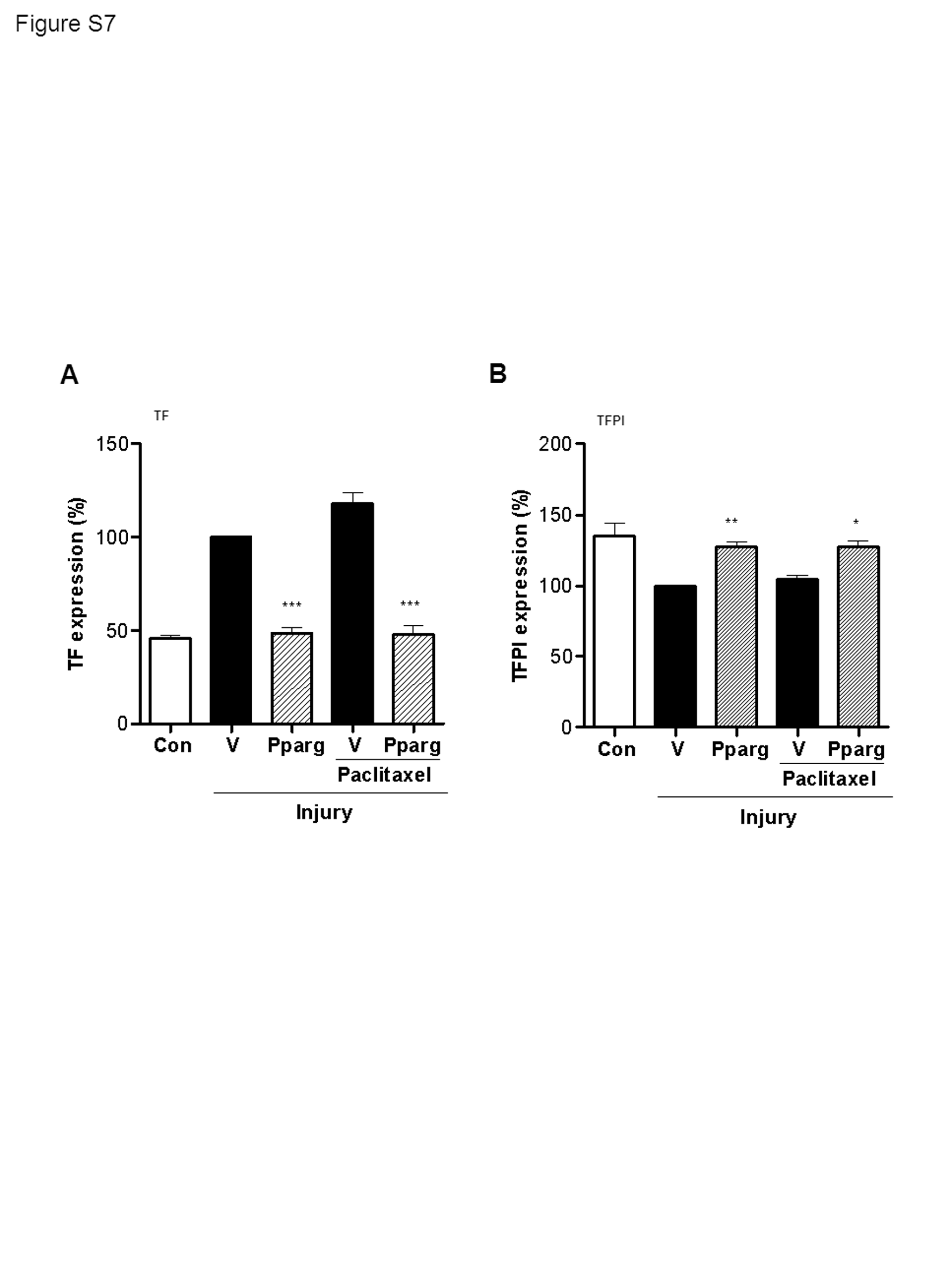

Supplement: Figure S7 — The changes of TF and TFPI expressions after PPAR-γ agonist treatment in vivo. (A–B) Bar graphs showed quantitative data for TF (A) and TFPI expressions (B) that are normalized to actin; Separate experiments were performed with 4 different rats per group. Data are presented as mean ± SEM. Values are given as percent of stimulation with balloon injury alone. *P<0.05, **P<0.01***P<0.0001 vs PPAR-γ agonist, or PPAR-γ agonist plus paclitaxel. (TIF) [file pone.0028327.s007.tif]

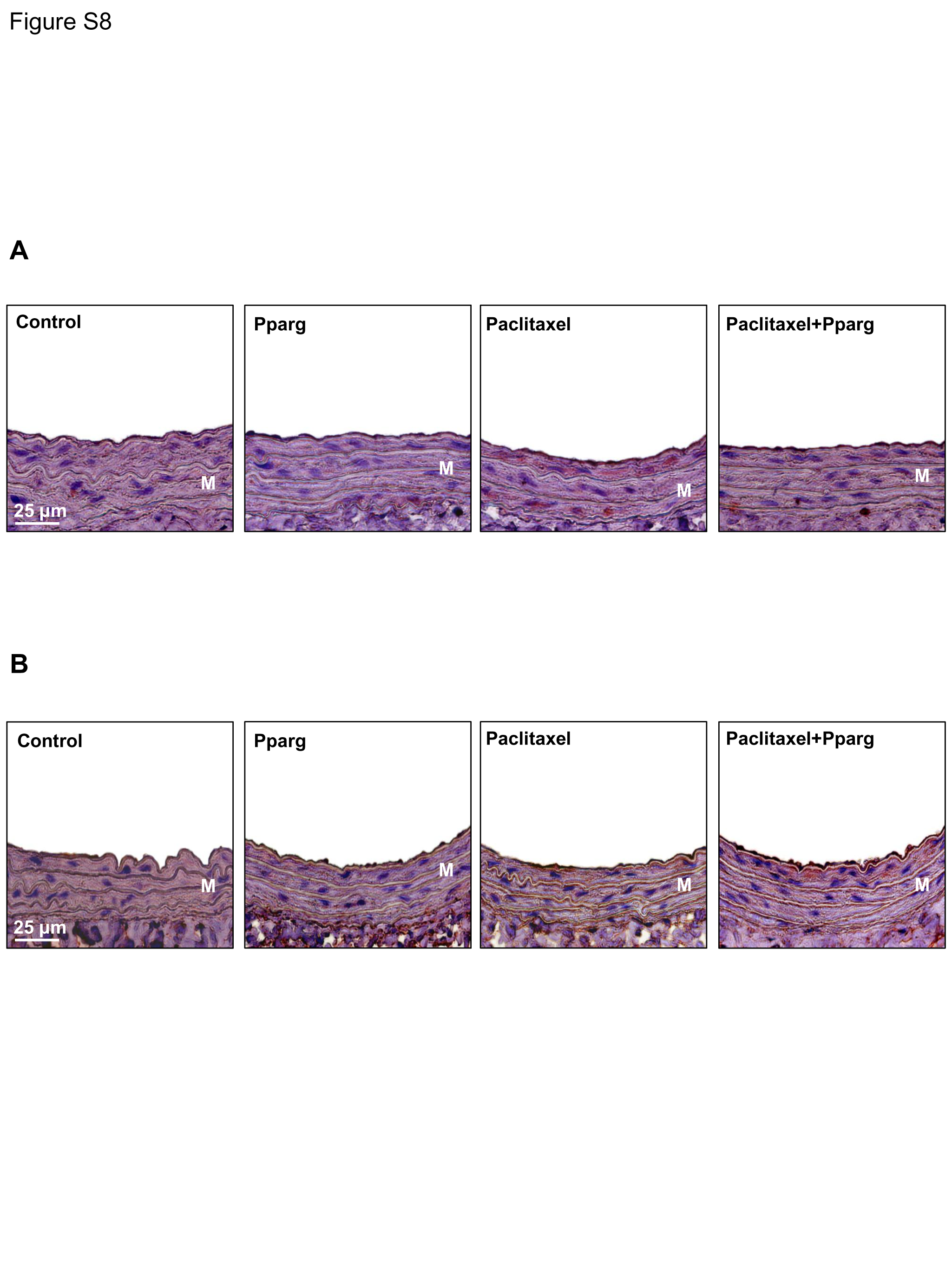

Supplement: Figure S8 — The effects of PPAR-γ agonist on TF and TFPI expressions in corresponding contralateral uninjured carotid arteries. (A–B) Representative images of IHC staining for TF or TFPI (hematoxylin-eosin stain, magnification x40). Paclitaxel without balloon injury slightly increased TF expression, which was reversed by the PPAR-γ agonist (A). With respect to TFPI expression, a minimal increase was observed in the uninjured artery treated with paclitaxel plus rosiglitazone, as compared to that treated with paclitaxel alone (B). Brownish-red indicates TF (upper panel) or TFPI protein (lower panel). (TIF) [file pone.0028327.s008.tif]
